# Methods for evaluating adverse drug event preventability in emergency department patients

**DOI:** 10.1186/s12874-018-0617-4

**Published:** 2018-12-04

**Authors:** Stephanie A. Woo, Amber Cragg, Maeve E. Wickham, David Peddie, Ellen Balka, Frank Scheuermeyer, Diane Villanyi, Corinne M. Hohl

**Affiliations:** 1 Pharmaceutical Sciences, Vancouver General Hospital, 855 West 12th Avenue, Vancouver, BC V5Z 1M9 Canada; 2 Department of Emergency Medicine, University of British Columbia, 855 West 12th Avenue, Vancouver, BC V5Z 1M9 Canada; 3 Centre for Clinical Epidemiology and Evaluation, Vancouver Coastal Research Institute, 828 West 10th Ave, Vancouver, BC V5Z 1M9 Canada; 4 School of Population and Public Health, University of British Columbia, 2206 East Mall, Vancouver, BC V6T 1Z9 Canada; 5 School of Communication, Simon Fraser University, Burnaby, BC Canada; 6 Division of Geriatrics, Department of Medicine, Vancouver General Hospital, 855 West 12th Avenue, Vancouver, BC V5Z 1M9 Canada; 7 Emergency Department, Vancouver General Hospital, 855 West 12th Avenue, Vancouver, BC V5Z 1M9 Canada

**Keywords:** Adverse drug event, Adverse drug reaction, Preventability, Preventable, Explicit review, Implicit review

## Abstract

**Background:**

There is a high degree of variability in assessing the preventability of adverse drug events, limiting the ability to compare rates of preventable adverse drug events across different studies. We compared three methods for determining preventability of adverse drug events in emergency department patients and explored their strengths and weaknesses.

**Methods:**

This mixed-methods study enrolled emergency department patients diagnosed with at least one adverse drug event from three prior prospective studies. A clinical pharmacist and physician reviewed the medical and research records of all patients, and independently rated each event’s preventability using a “best practice-based” approach, an “error-based” approach, and an “algorithm-based” approach. Raters discussed discordant ratings until reaching consensus. We assessed the inter-rater agreement between clinicians using the same assessment method, and between different assessment methods using Cohen’s kappa with 95% confidence intervals (95% CI). Qualitative researchers observed discussions, took field notes, and reviewed free text comments made by clinicians in a “comment” box in the data collection form. We developed a coding structure and iteratively analyzed qualitative data for emerging themes regarding the application of each preventability assessment method using NVivo.

**Results:**

Among 1356 adverse drug events, a best practice-based approach rated 64.1% (95% CI: 61.5–66.6%) of events as preventable, an error-based approach rated 64.3% (95% CI: 61.8–66.9%) of events as preventable, and an algorithm-based approach rated 68.8% (95% CI: 66.1–71.1%) of events as preventable. When applying the same method, the inter-rater agreement between clinicians was 0.53 (95% CI: 0.48–0.59), 0.55 (95%CI: 0.50–0.60) and 0.55 (95% CI: 0.49–0.55) for the best practice-, error-, and algorithm-based approaches, respectively. The inter-rater agreement between different assessment methods using consensus ratings for each ranged between 0.88 (95% CI 0.85–0.91) and 0.99 (95% CI 0.98–1.00). Compared to a best practice-based assessment, clinicians believed the algorithm-based assessment was too rigid. It did not account for the complexities of and variations in clinical practice, and frequently was too definitive when assigning preventability ratings.

**Conclusion:**

There was good agreement between all three methods of determining the preventability of adverse drug events. However, clinicians found the algorithmic approach constraining, and preferred a best practice-based assessment method.

**Electronic supplementary material:**

The online version of this article (10.1186/s12874-018-0617-4) contains supplementary material, which is available to authorized users.

## Background

Adverse drug events are unintended and harmful events related to medication use or misuse. They account for one in nine emergency department visits, and remain a leading cause of unplanned admissions and deaths [1–5]. Given the burden they place on patients, families, and the health system, developing and evaluating effective strategies for prevention is an international research and healthcare management priority [6].

Studies report highly variable rates of preventable adverse drug events [4, 7–11]. Prospective studies indicate that 28 to 80% of adverse drug events are considered preventable [12–19]. These differences may be due to variations in study design, heath setting, and patient populations, and also to the lack of standardized uniform preventability assessment methods [20–22]. This lack of consistency in definition and ascertainment of preventability undermines our ability to reproduce and compare results between studies, monitor trends over time, and meta-analyze research results.

The majority of adverse drug event preventability assessment methods described in the literature can be categorized into one of three central themes: (1) grounded in the concept of adherence to best medical practice [4, 22]; (2) rooted in error avoidance and identification of modifiable risk factors [23]; or, (3) application of an explicit algorithmic approach [24]. Our main objective was to assess these three different approaches for determining adverse drug event preventability in patients presenting to the emergency department by measuring the inter-rater agreement of reviewers when applying each method. Secondary objectives were to measure the inter-rater agreement between methods by comparing the consensus ratings for each method, and to explore their strengths and weaknesses using qualitative methods.

## Methods

### Study design

This was a mixed-methods sub-study of a multi-centre retrospective chart review investigating adverse drug event preventability (Hohl CM, Woo SA, Cragg A, et al: Repeat Adverse Drug Events to Outpatient Medications, submitted).

### Setting and population

We reviewed the medical and research records of all patients diagnosed with a suspect adverse drug event in one of three previously conducted prospective studies [2, 3, 5, 25]. The first study enrolled 1591 patients presenting to the emergency departments of two tertiary care hospitals, Vancouver General (VGH) and St. Paul’s Hospitals (SPH) in Vancouver, British Columbia, Canada, in 2008–2009, and derived tools to identify patients at high-risk of having an adverse drug event [3]. The second study enrolled 10,807 patients presenting to the emergency departments of VGH, Lions Gate Hospital (LGH), an urban community hospital in North Vancouver, British Columbia, and Richmond General Hospital (RGH), an urban community hospital in Richmond, British Columbia, between 2011 and 2013, and evaluated the impact of pharmacist-led medication review on health outcomes [5, 25]. The third study enrolled 1529 patients presenting to the emergency departments of VGH, LGH and the Ottawa Civic Hospital (OCH), an urban tertiary care hospital in Ottawa, Ontario, Canada, in 2014–2015, and validated the previously derived clinical decision rules [2].

### Inclusion/exclusion criteria

We have previously described the methodology of the original studies [2, 3, 5, 25]. Briefly, research assistants used a systematic selection algorithm to select and enrol a representative sample of emergency department patients. A clinical pharmacist and physician evaluated all enrolled patients for suspect adverse drug events at the point-of-care and documented the events in research and medical records. All cases in which the clinical pharmacist and physician diagnoses were concordant were considered final. An independent committee adjudicated all cases in which assessments were discordant or uncertain.

We included all participants from the primary studies who were diagnosed with a suspect adverse drug event and excluded patients for whom an alternative diagnosis was identified during the chart review process. We also excluded patients whose records could not be linked or retrieved from the provincial medication database, and those with illegible records.

### Chart review data collection methods

A clinical pharmacist (SW) and a physician (CH, DV or FS) conducted a structured explicit medical and research record review of all patients with suspect adverse drug events using standardized and piloted data collection forms (Additional file 1: Appendix A and Additional file 2: Appendix B). All four were blinded to study outcomes. They independently assessed the preventability of each adverse drug event using two different preventability definitions and one algorithm and categorized events as definitely preventable, probably preventable, or not preventable using each method [4, 22–24]. If preventability ratings between raters were discordant using the same method, reviewers discussed the case until reaching consensus. In cases of remaining uncertainty, a third reviewer adjudicated the case.

### Qualitative data collection methods

Researchers trained in qualitative methods (DP or EB) independently observed reviewers conducting preventability assessments and discussing discordant cases to gather insight into the review process and any challenges that reviewers experienced, and to identify common areas of uncertainty or discordance. We scheduled observations for ten, two- to three-hour data collection shifts at different hospital sites, with different observers to account for variation in patient populations, hospital sites, charting practices, and reviewers (e.g., reasoning, habits, assumptions, inter-personal relationships). We captured reflections about the reviewers’ assessments, and documented issues that they encountered. We also reviewed all free text comments made by reviewers in a field provided on our data collection form.

### Definitions

#### Adverse drug event

We defined an adverse drug event as “harm caused by the use or inappropriate use of a drug” [26]. Adverse drug events included events categorized as adverse drug reactions (i.e.*,* “noxious and/or unintended responses to medication which occurred despite appropriate drug dosage for prophylaxis, diagnosis or therapy of the indicating medical condition” [27]), drug interactions, supra- and sub-therapeutic doses, and events that occurred due to non-adherence, being on an ineffective drug, needing an additional drug (i.e.*,* in cases in which there was clear documentation at enrolment about an indication for the drug and lack of contraindication to it), cases due to errors, and drug withdrawals [28].

#### Best practice-based preventability assessment

A best practice-based preventability definition was developed by Hallas et al. [22], and subsequently modified for use in prospective studies [3, 4, 8]. It relies on reviewers’ clinical expertise, and defines adverse drug events as preventable if events were “avoidable by adhering to best medical practice, including inappropriate drug, dosage, route or frequency of administration of a drug for the patient’s clinical condition, age, weight or renal function; administration of a drug despite a known allergy, a previous adverse reaction to, or a drug interaction; noncompliance; laboratory monitoring not or inappropriately performed; prescribing or dispensing errors, or errors in drug administration” [4].

#### Error-based preventability assessment

We adopted the error-based preventability assessment definition from a definition provided by Health Canada, Canada’s drug regulatory organization. As such, preventable events include a medication error, as well as modifiable risk factors that were not addressed [23].

#### Algorithm-based preventability assessment

Schumock and Thornton developed an explicit algorithm to rate preventability based on prescribing and monitoring appropriateness for a drug (Table 1) [24]. Other authors have expanded and modified the algorithm to capture drug interactions, known treatments for adverse drug events, and preventative therapies [24, 29]. In this study, we expanded the algorithm during our pilot phase to capture medication-related errors and inappropriate re-exposures to culprit drugs, which were not captured in any previous iteration (Table 1) [4, 30–33].

**Table 1:** Modified Preventability Algorithm [24]

Definitely Preventable ADE	
1. Was there a history of allergy or previous reactions to the drug or drug class?	Yes/No/Uncertain
-If yes, was the re exposure inappropriate?^a^*	Yes/No/Uncertain
2. Was any drug involved inappropriate for the patient’s clinical condition?^a^	Yes/No/Uncertain
3. Was the dose, route or frequency of administration inappropriate for the patient’s age, weight or disease state?^a^	Yes/No/Uncertain
4.Was a toxic serum drug concentration (or laboratory monitoring test) documented?^a^	Yes/No/Uncertain
5. Was there a known treatment for the ADE? (eg. To prevent predictable drug side effects)^a^	Yes/No/Uncertain
Probably Preventable ADE	
6. Was required therapeutic drug monitoring or other necessary tests not performed?^b^	Yes/No/Uncertain
7. Was a drug interaction involved in the ADE?^b^	Yes/No/Uncertain
8. Was poor compliance involved in the ADE?^b^	Yes/No/Uncertain
9. Were preventative measures not prescribed or administered to the patient? (eg. Untreated indication?)	Yes/No/Uncertain
-If yes, were preventative measures not contraindicated?^b^*	Yes/No/Uncertain
Additional Criteria for ADE Preventability	
10. Was there an error in ADE diagnosis that contributed to the event persisting/getting worse?^a^*	Yes/No/Uncertain
11. Was there a delay in ADE diagnosis that contributed to the event persisting/getting worse?^b^*	Yes/No/Uncertain
12. Was there a failure to act on the result of monitoring or testing?^a^*	Yes/No/Uncertain
13. Were there errors in the transcription of the culprit drug(s) order?^a^*	Yes/No/Uncertain
14. Were there any errors in dispensing of the culprit drug(s) order?^a^*	Yes/No/Uncertain
15. Were there any errors in the administration of the culprit drug(s)?^a^*	Yes/No/Uncertain
16. Was a superior alternative treatment available (without contraindication) that is less likely to cause an ADE?^b^*	Yes/No/Uncertain
17. Was there any failure in communication that contributed to the ADE?^a^*	Yes/No/Uncertain
18. Was there any equipment failure that contributed to the ADE?^a^*	Yes/No/Uncertain
Automated preventability assessment based on algorithm	Definitely/Probably/Not

*Added to the modified Schumock and Thornton algorithm

^a^If yes, event is rated as “definitely preventable”

^b^If yes, event is rated as “probably preventable”

### Analysis

#### Quantitative

We used descriptive statistics to describe all enrolled patients and adverse drug events. We reported proportions with 95% confidence intervals (95%CIs) or means or medians with appropriate measures of variance depending on the data distribution. We used consensus preventability ratings to calculate the proportion of adverse drug events deemed definitely, probably and not preventable for each preventability assessment instrument. We calculated the proportion of preventable events by grouping definitely and probably preventable events and dividing this number by the number of all events for each instrument. To evaluate inter-rater reliability of each method between clinicians, we compared the original pharmacist and physician ratings for each adverse drug event, by instrument. To evaluate between-instrument reliability, we compared the consensus rating for each adverse drug event between instruments. In each case, we calculated kappa scores with 95% confidence intervals for definitely and probably preventable adverse drug events versus non-preventable events and for definitely preventable versus probably preventable events. We used the pharmacist’s rating to determine which criteria from the algorithm-based preventability assessment most frequently contributed to a definitely or probably preventable rating, by calculating the proportion of criteria deemed positive, over all events.

#### Qualitative

We coded field notes from our observations and free text comments from reviewers using qualitative data analysis software (NVivo Version 11, QSR International, Doncaster, Victoria, Australia). We generated thematic summaries that were verified and refined with reviewers’ perspectives. The purpose of this approach was to generate detailed accounts of the preventability assessment process that resonated with reviewers [34]. We concluded observation shifts when they no longer yielded novel insights. We used qualitative findings to provide context to our quantitative results.

## Results

### Quantitative results

**Fig. 1:**
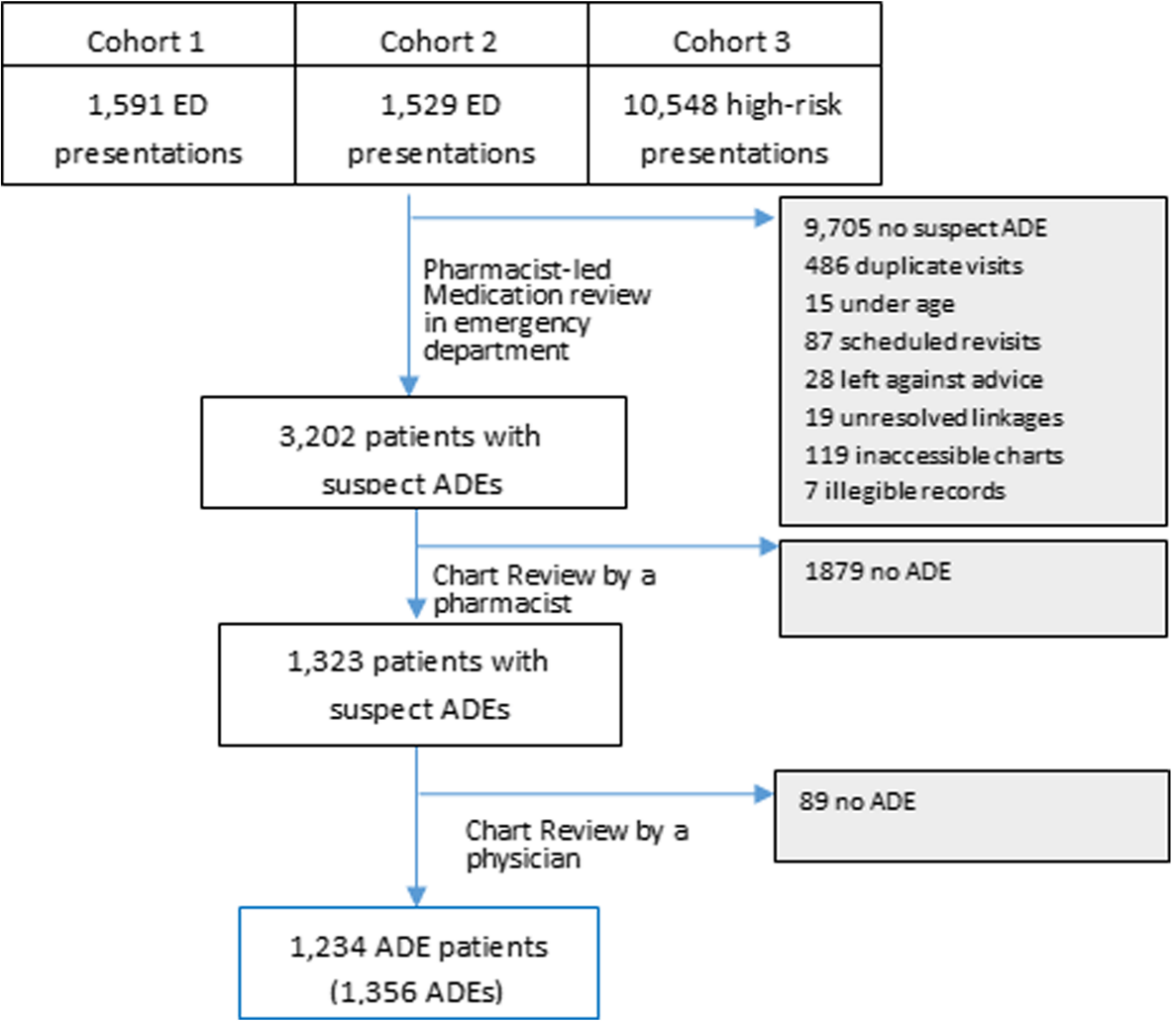
Flow diagram of patients through the study

We reviewed the charts of 3202 patients with suspect adverse drug events, of whom 1234 were diagnosed with at least one adverse drug event (Fig. 1). We identified 1356 adverse drug events. Reviewers rated 64.1% (95% CI 61.5–66.6) of adverse drug events as definitely or probably preventable by the best practice-based assessment, 64.3% (95% CI 61.8–66.9) by the error-based assessment and 68.6% (95% CI 66.1–71.1) by the algorithm-based assessment (Table 2). The proportion of events rated as definitely preventable was highest using the algorithm-based assessment (45.2, 95% CI 42.6–47.9).

**Table 2:** Consensus preventability ratings of 1356 adverse drug events, by assessment method

Preventability Rating	Best Practice-Based [4]	Error-Based [23]	Algorithm-Based [24]
Definitely or probably preventable, n (%, 95 CI)	869 (64.1, 61.5–66.6)	930 (68.5, 66.1–71.1)	873 (64.3, 61.8–66.9)
Definitely, n (%, 95 CI)	87 (6.4, 5.1–7.7)	93 (6.9, 5.5–8.2)	613 (45.2, 42.6–47.9)
Probably, n (%, 95 CI)	782 (57.7, 55.0–60.3)	780 (57.5, 54.9–60.2)	317 (23.4, 21.1–25.6)
Not preventable, n (%, 95 CI)	487 (35.9, 33.4–38.5)	483 (35.6, 33.1–38.2)	426 (31.4, 28.9–33.9)

#### Agreement between reviewers and assessment method

The inter-rater agreement of each instrument applied by different clinicians ranged from 0.53 (95% CIs 0.48–0.59) to 0.55 (0.50–0.60) when determining definitely and probably preventable events versus non-preventable events, with overlapping confidence intervals, indicating no significant differences (Table 3). The inter-rater agreements between clinicians for discerning definitely and probably preventable events were more modest (Table 4).

**Table 3:** The inter-rater agreement between reviewers determining definitely and probably preventable events versus non-preventable events, by assessment method

Preventability Assessment	Inter-rater agreement Kappa (95% CIs) *n* = 1356
Best Practice-Based [4]	0.53 (0.48–0.59)
Error -Based [23]	0.55 (0.50–0.60)
Algorithm-Based [24]	0.55 (0.49–0.60)

**Table 4:** The inter-rater agreement between reviewers in determining definitely versus probably preventable events, by assessment method

Preventability Definition	Inter-rater Agreement Kappa (95% CIs)
Best Practice-Based [4] (*n* = 869)	0.33 (0.23–0.44)
Error -Based [23] (*n* = 873)	0.30 (0.20–0.40)
Algorithm-Based [24](*n* = 930)	0.40 (0.33–0.46)

The consistency in ratings between the three preventability instruments was excellent, with kappa scores ranging from 0.88 (95% CI 0.85–0.91) to 0.99 (95% CI 0.98–1.00; Table 5). Inter-instrument agreement was highest when comparing the best-practice and error-based definitions.

**Table 5:** Agreement between assessment methods, comparing preventable versus non-preventable adverse drug events, using consensus ratings

Consensus Ratings by Definition	Instrumental Agreement Kappa (95% CIs) *n* = 1356
Best Practice-Based [4] vs. Algorithm-Based [24]	0.88 (0.85–0.91)
Algorithm-Based [24] vs. Error -Based [23]	0.89 (0.86–0.91)
Best Practice-Based [4] vs. Error -Based [23]	0.99 (0.98–1.00)

### Qualitative results

Reviewers observed that the algorithm-based assessment was often unable to account for the realities of day-to-day medication use and clinical practice and noted that the algorithm frequently overestimated the preventability of an event. Reviewers commonly felt that an assertion of “definitely” preventable was problematic without knowing other circumstances of the patient’s care, their interaction with their care providers and the system, the frequency of the patient’s monitoring, and prior providers’ decision-making. The algorithm-based assessment also asked reviewers to determine the preventability of multi-factorial events based on one single criterion. The questions most frequently producing “definitely” preventable ratings discordant with the other assessments were re-exposures to the same culprit medication (which may have been appropriate in some clinical scenarios), and the presence of a toxic drug serum concentration or abnormal lab results (which reviewers felt did not equate to the events being always definitely preventable; Table 6). For example, reviewers noted that some patients on warfarin came to the emergency department because their international normalized ratio (used to monitor warfarin therapy) was outside of its therapeutic range yielding a “definitely” preventable assessment using the algorithm-based assessment, even though reviewers may have felt that the abnormal results may have been unpredictable (e.g., the result of an intermitted illness) and therefore not definitively preventable.

**Table 6:** Questions in the algorithm-based approach for which a YES answer automatically resulted in a definitely preventable rating without the option of modifying it to probably preventable

Schmock and Thornton algorithm questions (response)	Frequency, n (%) *n* = 508
Was there a history of allergy or previous reactions to the drug or drug class? (YES) Was the re-exposure appropriate? (NO)	148 (29.1)
Was a toxic serum drug concentration (or laboratory monitoring test) documented? (YES)	144 (28.4)
Was any drug involved inappropriate for the patient’s clinical condition? (YES)	78 (15.4)
Was the dose, route or frequency of administration inappropriate for the patient’s age, weight or disease state? (YES)	66 (13.0)
Was there a known treatment for the ADE? (e.g., to prevent predictable drug side effects?) (YES) Was a known treatment not prescribed or administered to the patient? (YES) Was the known treatment contraindicated? (NO)	38 (7.5)
Was there any failure in communication that contributed to the ADE? (YES)	17 (3.4)
Was there a failure to act on the results of monitoring or testing? (YES)	11 (2.2)
Were there any errors in the administration of the culprit drug(s)? (YES)	5 (1.0)
Was there an error in ADE diagnosis that contributed to the event persisting/getting worse? (YES)	1 (0.2)

The best practice-based preventability assessment asked reviewers to rely on their clinical impression of the adverse drug event. The notion of what constituted ‘best medical practice’ often invoked reviewer discussions when adjudicating according to the best-practice definition. Reviewers with differing perspectives often attributed responsibility for preventable events to different levels of or roles within the health system. For example, reviewers argued preventability may be located at the level of patients (e.g.*,* patient-level medication adherence), providers (e.g.*,* prescribing practices), or the system (e.g.*,* informational discontinuity between healthcare sectors).

Reviewers noted that best practice was not always clear-cut, especially when only having access to the medical record at a distance from the event. Definitions of best practice varied depending on the professional training and perspective of the reviewer, and reviewers referred to the clinical reality, in which optimizing treatment could be a matter of trial and error, where many competing factors required consideration, and where treatment guidelines may differ and even contradict themselves. Consequently, reviewers generally preferred to rate events as probably, rather than definitely preventable, which made the best practice approach more favorable than the algorithm-based approach.

Reviewers noted that they often had to make categorizations based on the information in the patient’s chart, which could be incomplete, vague, or offered conflicting information. For example, if information on blood work monitoring was unavailable in the medical record system because it had been performed in a different institution, reviewers were unable to judge where a relevant laboratory value was outside a normal range, and how often it had been monitored.

## Discussion

We sought to compare three methods of assessing adverse drug event preventability and explore their strengths and weaknesses. All methods to assess preventability found approximately two-thirds of adverse drug events to be preventable, with excellent overall agreement between assessments.

The shortcomings of the algorithm and error-based assessments lead reviewers to prefer the best practice approach. The principal weakness of the algorithm-based assessment was its reductionist approach in determining preventability. Observational studies assessing quality of care indicate other similar algorithm-based assessments fail to distinguish small lapses in specific measures from the overall context of adequate care [35–37]. These assessments have a limited capacity to capture the complexities of care [37]. The reductionist nature of the algorithm-based assessment also highlights the effect of hindsight bias [38], which may influence preventability ratings in light of an adverse drug event. For example, a typically benign drug interaction is considered “probably preventable” only in the occurrence of an adverse drug event. Similarly, discrepancies between the best practice and error-based assessments may be attributed to the error-based approach being more critical of errors and risk factors found outside of the realms of best practice, in part due to hindsight bias [39, 40]. As such, reviewers found the best practice approach the most appropriate because it allowed reviewers to rate the event according to multiple factors, and the context of the circumstances leading up to the event.

For rating the preventability of adverse drug events, a more suitable classification may be a binary rating, in which preventable events comprise of probably and definitely preventable events versus unlikely or non-preventable events. We found higher agreement between reviewers rating events as preventable or not preventable, which suggests this binary classification to be more reliable than differentiating between definitely and probably preventable events. A binary rating system would also negate some of the flaws of the assessments that we studied, including situations where reviewers found the algorithm to overestimate the deviation from adequate care, a common observation in other retrospective studies that used other criteria-based approaches to assess quality of care [36, 37, 41, 42]. By merging definitely and probably preventable events, the magnitude of overestimation in preventability that occurs due to hindsight bias may be mitigated.

The inter-rater reliability of preventability between clinicians was good for all assessment methods when used in a binary approach. Individual and professional biases have previously been implicated in poor inter-rater reliability when assessing quality of care, which is consistent with our observation that individual and professional backgrounds oriented reviewers to how they perceived an event resulting in different ratings [35, 43, 44]. We believe there is value in using a second reviewer to rate the preventability of adverse drug events, and to ask reviewers to discuss discordant ratings until reaching consensus to minimize these biases by reflecting the experiences and perspectives of multiple clinicians.

The primary limitation of our findings is the generalizability of their application. The best practice and error-based assessments provide greater context in the overall assessment of preventability. However, assessing the global impression of care leading to an adverse drug event requires a high level of clinical experience and expertise [45]. In a setting where resources only allow for students or care providers with a limited clinical background, the algorithm-based assessment may be appropriate if “definitely” and “probably” preventable categories are combined to a probably preventable category to create a more global view of preventability, and reflect the remaining uncertainty in any retrospective assessment.

## Conclusion

A standardized assessment of adverse drug event preventability coordinates our efforts to understand areas of care where interventions may have a greater likelihood of reducing adverse drug event burden on patients and the health system. We found good agreement of preventability between different reviewers and assessments, and recommend use of a best-practice definition assessed by two or more clinicians with differing perspectives to produce a conservative preventability estimate. Our findings also indicate that there is little merit in categorizing events as definitely versus probably preventable; we suggest that these categories be collapsed together in future assessments of adverse drug event preventability.

## Additional files

Additional file 1: Appendix A. Data Collection Form for Preventability Assessments. (DOCX 18 kb)

Additional file 2: Appendix B. Data Collection Form for Physician Preventability Assessments and Consensus Ratings. (DOCX 19 kb)

## Abbreviation

ADE: Adverse drug event
