# Supplementary material for: Methods for evaluating adverse drug event preventability in emergency department patients
Source: BMC Med Res Methodol. 2018 Dec 4;18:160. doi: 10.1186/s12874-018-0617-4 (PMC6280499; doi:10.1186/s12874-018-0617-4)
Supplement: Supplementary file 2 — Appendix B. Data Collection Form for Physician Preventability Assessments and Consensus Ratings. (DOCX 19 kb) [file 12874_2018_617_MOESM2_ESM.docx]

**Appendix B.** Data Collection Form for Physician Preventability Assessments and Consensus Ratings.

| **Physician Information** | |
| --- | --- |
| Physician Surname: | Select from dropdown list |
| Patient Study ID | Select from dropdown list |
| ADE number | ADE1/ADE2/ADE3 |
|  | |
| **ADE information** | |
| Culprit Drug(s): | Load automated from studyID and ADE# |
| Sign(s)/Symptom(s): | Load automated from studyID and ADE# |
| Diagnosis: | Load automated from studyID and ADE# |
|  | |
| **Physician ADE Assessment** | |
| Do you think that this patient was suffering from an adverse drug event? | Yes/No/Uncertain |
| - If uncertain or no, please indicated why and include your working diagnosis: |  |
|  | |
| **Physician Preventability Assessment #1** | |
| Based on the following ADE preventability definition, please rate the preventability of the ADE.  “Avoidable by adhering to best medical practice. This includes: inappropriate drug, dosage, route or frequency of administration for the patient’s clinical condition, age, weight or renal function; administration despite a known allergy, a previous adverse reaction to, or a drug interaction with this drug; noncompliance; laboratory monitoring not or inappropriately performed; prescribing or dispensing errors, or errors in drug administration.” | *Definitely/Probably/Not* |
|  | |
| **Physician Preventability Assessment #2** | |
| Based on the following ADE preventability definition, please rate the preventability of the ADE.  “Preventable events include medication errors as well as modifiable risk factors that were not addressed.” | *Definitely/Probably/Not* |
|  | |
| **Physician Preventability Assessment #3** | |
| 1. Was there a history of allergy or previous reactions to the drug or drug class? | Yes/No/Uncertain |
| - If yes, was the re-exposure appropriate? | Yes/No/Uncertain |
| 1. Was any drug involved inappropriate for the patient’s clinical condition? | Yes/No/Uncertain |
| 1. Was the dose, route or frequency of administration inappropriate for the patient’s age, weight or disease state? | Yes/No/Uncertain |
| 1. Was a toxic serum drug concentration (or laboratory monitoring test) documented? | Yes/No/Uncertain |
| 1. Was there a known treatment for the ADE? (eg. To prevent predictable drug side effects?) | Yes/No/Uncertain |
| 1. Was required therapeutic drug monitoring or other necessary tests not performed? | Yes/No/Uncertain |
| 1. Was a drug interaction involved in the ADE? | Yes/No/Uncertain |
| 1. Was poor compliance involved in the ADE? | Yes/No/Uncertain |
| 1. Were preventative measures not prescribed or administered to the patient? (eg. Untreated indication?) | Yes/No/Uncertain |
| - If yes, were preventative measures contraindicated? | Yes/No/Uncertain |
| 1. Was there an error in ADE diagnosis that contribute to the event persisting/getting worse? | Yes/No/Uncertain |
| 1. Was there a delay in ADE diagnosis that contributed to the event persisting/getting worse? | Yes/No/Uncertain |
| 1. Was there a failure to act on the result of monitoring or testing? | Yes/No/Uncertain |
| 1. Were there errors in the transcription of the culprit drug(s) order? | Yes/No/Uncertain |
| 1. Were there any errors in the dispensing of the culprit drug(s) order? | Yes/No/Uncertain |
| 1. Were there any errors in the administration of the culprit drug(s)? | Yes/No/Uncertain |
| 1. Was a superior alternative treatment available (without contraindication) that is less likely to cause an ADE? | Yes/No/Uncertain |
| 1. Was there any failure in communication that contributed to the ADE? | Yes/No/Uncertain |
| 1. Was there any equipment failure that contributed to the ADE? | Yes/No/Uncertain |
|  | |
| Automated preventability assessment based on algorithm | *Definitely/Probably/Not* |
